# Molecular Evolution of the Glutathione S-Transferase Family in the *Bemisia tabaci* Species Complex

**DOI:** 10.1093/gbe/evaa002

**Published:** 2020-01-23

**Authors:** Ofer Aidlin Harari, Diego Santos-Garcia, Mirit Musseri, Pnina Moshitzky, Mitulkumar Patel, Paul Visendi, Susan Seal, Rotem Sertchook, Osnat Malka, Shai Morin

**Affiliations:** e1 Department of Entomology, The Hebrew University of Jerusalem, Rehovot, Israel; e2 Natural Resources Institute, University of Greenwich, Kent, United Kingdom; e3 Consultant, Protein Modelling, Gedera, Israel

**Keywords:** molecular evolution, detoxification, positive selection, sap-feeding insect, species complex, glutathione S-transferase

## Abstract

The glutathione S-transferase (GST) family plays an important role in the adaptation of herbivorous insects to new host plants and other environmental constrains. The family codes for enzymes that neutralize reactive oxygen species and phytotoxins through the conjugation of reduced glutathione. Here, we studied the molecular evolution of the GST family in *Bemisia tabaci*, a complex of >35 sibling species, differing in their geographic and host ranges. We tested if some enzymes evolved different functionality, by comparing their sequences in six species, representing five of the six major genetic clades in the complex. Comparisons of the nonsynonymous to synonymous substitution ratios detected positive selection events in 11 codons of 5 cytosolic GSTs. Ten of them are located in the periphery of the GST dimer, suggesting a putative involvement in interactions with other proteins. Modeling the tertiary structure of orthologous enzymes, identified additional 19 mutations in 9 GSTs, likely affecting the enzymes’ functionality. Most of the mutation events were found in the environmentally responsive classes Delta and Sigma, indicating a slightly different delta/sigma tool box in each species. At a broader genomic perspective, our analyses indicated a significant expansion of the Delta GST class in *B. tabaci* and a general association between the diet breadth of hemipteran species and their total number of GST genes. We raise the possibility that at least some of the identified changes improve the fitness of the *B. tabaci* species carrying them, leading to their better adaptation to specific environments.

## Introduction

The interactions of insects with their host plants and their necessity to adapt to utilizing them as a food source, mating and oviposition sites, are hypothesized to be a major driving force in the evolution of herbivorous insects (Mitter et al. 1988; Simon et al. 2015; Gouin et al. 2017; Kergoat et al. 2017). During the adaptation process, it is assumed that natural selection forces act among others, on individual genes and/or gene families that are required for successful host utilization. Among these families are chemosensation receptors used for host finding, salivary effectors for manipulating the plant defense responses, primary metabolic enzymes for digestion, and detoxification enzymes for coping with the plant toxic defense chemistry (Després et al. 2007; Mithöfer and Boland 2012; Simon et al. 2015; Kergoat et al. 2017; Boulain et al. 2018).

The detoxification of plant toxic defense compounds occurs in herbivorous insects in three phases. First, phase I enzymes from the families P450 monooxygenases (P450s) and carboxylesterases (COEs) oxidize, hydrolyze, and/or reduce the toxic substrate. Then, phase II enzyme families like Glutathione S-transferases (GSTs), sulfotransferases and, Uridine 5'-diphospho-glucuronosyltransferases catalyze the conjugation of substrate with hydrophilic groups such as glutathione, sulfate, or sugars, making the substrate less reactive and more polar, and therefore, more soluble and easier to excrete. Finally, in phase III, active export of the conjugated substrates out of the cell is performed by Adenosine triphosphate (ATP) binding cassette transporters (ABC transporters) (utilizing ATP) (Jakoby and Zieglers 1990; Liston et al. 2001; Després et al. 2007; Broehan et al. 2013).

The phase II GST enzymes family is present in all aerobic organisms and is divided into two different groups, microsomal enzymes and cytosolic enzymes. The microsomal GSTs form trimers in the membranes (Lundqvist et al. 1992; Lee and DeJong 1999), whereas the cytosolic GSTs are functional as homo/hetero-dimers. As second-phase enzymes, GSTs often work on the modified products of P450s or COEs, by catalyzing the conjugation of the tripeptide glutathione (glutamate, glycine, and cysteine) to various hydrophobic–electrophilic substrates. This is performed by binding reduced glutathione at the glutathione binding site (G-site), located at the amino end of the protein, lowering the thiol group p*K*_a_, which makes it more reactive. The hydrophobic substrate binds at the substrate binding site (SBS, H-site), located at the carboxyl end of the protein and adjacent (sterically) to the bound glutathione. In this way, the enzyme catalyzes the nucleophilic attack by the glutathione and its conjugation to the substrate, making the substrate less reactive and more soluble and therefore easier to excrete (Atkins et al. 1993; Sheehan et al. 2001; Enayati et al. 2005). A third conserved domain is the GSTs dimerization site (dimer interface), which includes the residues that form bonds between the subunits of the enzymes (Sinning et al. 1993) (supplementary fig. S1, Supplementary Material online).

Relatively little is known on the function/s and roles of microsomal GSTs in insects. Null mutations in the *microsomal GST I* (*MGST-I*) of *Drosophila melanogaster* (Diptera: Drosophilidae) were found to reduce the flies life span (Toba and Aigaki 2000). Microsomal GSTs of *Nilaparvata lugens* (Hemiptera: Delphacidae) showed upregulated expression in response to different insecticides. Moreover, knockdown by RNA interference of *GSTm2* increased the mortality of *N. lugens* instars during exposure to organophosphates (Zhou et al. 2013). On the other hand, the cytosolic GSTs are well characterized in insects and are divided into six classes, which differ in their SBS domain sequences: Theta, Zeta, Omega, Sigma, Delta, and Epsilon (Shou-min 2012). The classes Theta, Zeta, and Omega are generally conserved among organisms from distant phylogenetic lineages and were shown to play a role in primary metabolism such as catabolism of phenylalanine and tyrosine and catalysis of reductase reactions (Frova 2003; Oakley 2011; Shou-min 2012). Enzymes in the Sigma class are also present in a wide range of organisms and were shown to play a role in protection from oxidative stress (Singh et al. 2001; Sawicki et al. 2003). The Delta and Epsilon GSTs are unique to insects (Friedman 2011). These two classes were shown to be involved in the neutralization of environmental toxins and to play an important role in the adaptation of insects to their environment (Enayati et al. 2005; Ranson and Hemingway 2005; Shou-min 2012). The number of Sigma, Delta, and Epsilon GSTs varies between species/lineages mostly due to duplication events that seem to occur independently in each species (Friedman 2011).

The importance of cytosolic GSTs in the detoxification of plant toxins in herbivorous insects is well documented. Feces of larvae of various generalist lepidopteran species, fed on a diet containing isothiocyanates (metabolites that are formed when glucosinolates producing plants are injured), were found to contain products of the conjugation of these derivatives with glutathione (Schramm et al. 2012). In *Spodoptera litura* (Lepidoptera: Noctuidae), injection of dsRNA against the *GST epsilon 1* gene (*Slgste1*) decreased the insect’s ability to detoxify toxic compounds when feeding on *Brassica juncea* leaves or diet containing isothiocyanates (Zou et al. 2016). In the aphid *Myzus persicae* (Hemiptera: Aphididae), GSTs were found to be overexpressed when aphids were fed on a diet containing glucosinolates and isothiocyanates (Francis et al. 2005). Further investigation revealed that aliphatic glucosinolates pass through the aphid gut intact, whereas indolic glucosinolates are mostly hydrolyzed and are conjugated with ascorbate, glutathione, and various amino acids (Kim and Jander 2007; Kim et al. 2008). In *D. melanogaster*, GstD1 has an exceptionally broad substrate specificity and is one of the most efficient GSTs in catalyzing the conjugation of isothiocyanates to glutathione in vitro (Saisawang et al. 2012). Moreover, GstD1 orthologous enzymes can efficiently metabolize isothiocyanates in *Scaptomyza flava* and *Scaptomyza nigrita*, two species belonging to the genus *Scaptomyza* (an herbivorous lineage nested within the genus *Drosophila*) that feed on Brassicaceae (Whiteman et al. 2012; Gloss et al. 2014). Silencing genes coding for GST and COE enzymes in *N. lugens* increased the susceptibility of the insect to ferulic acid, a methoxy-hydroxylated derivative of cinnamic acid present in rice (Yang et al. 2017).

Here, we studied the GST family of the phloem-feeding insect model *Bemisia tabaci* (Hemiptera: Aleyrodidae). *Bemisia tabaci* is a complex of more than 35 cryptic species, divided into at least 6 main genetic groups (clades): Asia I/Australia, China, Asia II, sub-Saharan Africa (SSA), New-World, and Africa/Middle East/Asia Minor (De Barro et al. 2011). The divergence of the *B. tabaci* complex most likely occurred mainly through allopatric forces during migrating waves from SSA into new territories and continental drift (Boykin et al. 2013). However, data also suggest that within genetic groups, species might be very young (Hsieh et al. 2014; Santos-Garcia et al. 2015), allowing the detection of recent episodes of gene duplication and positive selection (see below). The species differ from one another in various biological traits such as virus transmission efficiency, insecticide resistance, fecundity, dispersal, and mating behaviors (Costa and Brown 1991; Bedford et al. 1994; Perring 2001). Moreover, there is a great variation in the documented host range of the different species, which might indicate differences in the species’ ability to detoxify secondary toxic metabolites produced by the various host plants (Malka et al. 2018).

The idea that the dynamics (“birth‐and‐death” processes and neofunctionalization) of gene families, known to be involved in host finding and food digestion and detoxification, might be associated with diet breadth in insect herbivores is not new. Rane et al. (2016, 2019) combined data on the size of the P450, COE, and GST gene families in 160 insect species from six orders, with data on the species’ diet breadth. In general, 2- to 4-fold variation was found across the species. Clear relationship between the numbers of detoxification genes and host use was found in the hemipteran and lepidopteran species analyzed (Rane et al. 2019). Following this, several studies on lepidopteran species varying in their host breadth provided strong support for a positive correlation between significant expansions of gene families/subfamilies involved in chemosensation (almost all gustatory receptors), digestion (midgut proteases and lipases) and detoxification (mainly P450s, COEs and GSTs), and polyphagy. Furthermore, these studies brought evidence that many of the expanded clusters show rapid amino acid sequence divergence and modified expression patterns that likely contribute directly to their greater functional versatility (Calla et al. 2017; Gouin et al. 2017; Pearce et al. 2017). Data from hemipteran species are less detailed. A recent study on five aphid species, differing in their host range, did not identify clear correlation between the family size of detoxification related genes and the plant host range of the analyzed species (Quan et al. 2019). Here, we expended these analyses by conducting a detailed comparison of the GST family size versus host range across the hemipteran order. In addition, we looked for evidence both for gene duplication and the acquisition of new functionalities in the GST family within the *B. tabaci* complex.

The only well annotated genome so far in the *B. tabaci* species complex belongs to the MEAM1 (Middle East/Asia Minor 1) species and was predicted to harbor 24 GST genes: 14 Delta, 5 Sigma, 2 Zeta, 2 Microsomal, and 1 Omega (Chen et al. 2016). Most of the studies in *B. tabaci* on the possible involvement of the GST family in the interactions of the insect with its plant hosts focused so far on gene expression changes during shifts to diets containing different levels of glucosinolates and/or flavonoids (Xie et al. 2011; Alon et al. 2012; Elbaz et al. 2012; Halon et al. 2015; Eakteiman et al. 2018; Malka et al. 2018). In the work reported here, we compared the GST coding sequences of six *B. tabaci* species, representing five of the six major genetic clades of the complex. We searched for signatures of adaptive evolution (changes in enzyme/s functionality) that might reflect differences in the ability of the species to utilize different environments and diets (Futuyma 1997; Hahn 2008). First, we recovered the sequences of all GSTs from the six studied species. Then, we applied several tests, all based on differences in the ratio of nonsynonymous to synonymous substitutions (d*N*/d*S*), to detect natural selection signatures, both at the whole coding sequence and at the codon (sites) levels. Finally, we used three-dimensional structure modeling to identify mutations with a potential to change important properties of the catalytic pocket, such as volume, shape, hydrophobicity of the surface, and affinity of the SBS to the substrate.

## Materials and Methods

### *Bemisia tabaci* and Host Plant Species

Six species of *B. tabaci*, representing five different geographical clades, were selected for analyses: SSA1-SG3 (SSA genetic group, subgroup 3, collected in Tanzania in 2013), ASIAII-1 (Asia II genetic group, species 1, collected in Pakistan in 2013), New-World 2 (hereafter NW2) (American genetic group species 2, collected in Brazil in 2013), MEAM1 (Middle East-Asia Minor, species 1) and MED-Q2 (Mediterranean Q, subgroup 2) (Africa/Middle East/Asia minor genetic group, collected in Israel in 2003), and Australia II (hereafter Australia) (Australia genetic group, species 2, collected in Australia in 2010). The identity of the six species was previously verified using their mitochondrial cytochrome C oxidase subunit I gene (*mtCOI*) DNA sequences (Malka et al. 2018). Individuals were collected for RNA purification from colonies that were reared on various host plants (commercial varieties of watermelon, cotton, pepper, and Brussels sprout) under standard conditions of 28 ± 2 °C, 60% humidity, and a 14:10 h light:dark cycle.

### Sequencing the *B. tabaci* GST Family

The research started previous to the publication of the MEAM1 genome (Chen et al. 2016). Therefore, raw data from several *B. tabaci* transcriptomes were downloaded in order to search for transcripts of GST genes. MEAM1: SRX022878, SRA036954, SRR835757; MED-Q1: SRX018661, SRR316271, SRR835756, PRJNA293094; and Asia II-3: SRR062575. Contigs annotated as GST were collected from all transcriptomes and their sequence identity was compared using BlastN. Contigs with more than 95% identity were considered as transcripts of the same gene (e.g., isoforms or allelic variations) and their alignment (MAFFT v7.215, L-INS-i algorithm, Katoh and Standley 2013) was used to obtain a consensus sequence. The consensus sequences were used to design Polymerase Chain Reaction (PCR) primers adjacent to the assumed start and stop codons of each gene for amplification (supplementary table S1, Supplementary Material online).

Pools of ∼200 individuals from each species were collected and RNA extraction was conducted (ISOLATE II RNA Mini kit, Bioline). cDNA synthesis was done using RevertAid H Minus Reverse Transcriptase (Thermo Scientific), according to the manufacturer instructions. This was followed by PCR amplification, using the relevant primer set of each GST and the high-fidelity enzyme KAPA HiFi (PCR kit, Biosystems). The PCR products were run in 1% agarose gel, recovered (Zymoclean Gel DNA Recovery kit, Zymo Research), cloned into pJET plasmids (CloneJET PCR Cloning kit, Thermo Scientific), and transformed into DH5α competent cells (Real Biotech Corporation). At least four colonies were selected by colony-PCR from each insert (SuperTherm Taq DNA Polymerase, Roche). Plasmids were purified using the ZR Plasmid miniprep – classic kit (Zymo Research, D4016) and sent for sequencing (Hy-Labs, Israel or Macrogen, Korea). In cases of failure to amplify the transcript of a specific GST from one or more species, the aforementioned transcriptomes and/or our recently published transcriptomic data (NCBI short read archive SRP127757) were used to design species/gene-specific primers. Finally, one representative sequence of each GST gene in each analyzed species was selected for further analysis. In cases where polymorphisms were detected, the majority rule was applied. BlastN reciprocal best hit (Camacho et al. 2009) was conducted to identify the orthologous GST gene in the MEAM1 genome (Chen et al. 2016). Then, the nomenclature suggested by Chen et al. (2016) was applied.

### Phylogenetic Analysis

*Drosophila melanogaster* GST sequences were obtained using the FlyBase server (http://flybase.org, last accessed January 23, 2020), by feeding “GST” in the search line. The *D. melanogaster* GST sequences are well annotated and can be used for accurate classification of homologous genes. To explore homology between enzymes of different hemipterans and to increase the depth of the data for more precise calculations, two hemipteran species were also included: *Trialeurodes vaporariorum* and *M. persicae.* In the case of *T. vaporariorum*, all transcripts annotated as a GST in the published transcriptome (SRA024353.1) were included in our analysis (Karatolos et al. 2011). *Myzus* *persicae* GST sequences were obtained by the procedure described in the section below. Sequences were aligned (see Supplementary alignments) using MAFFT v7.215 (L-INS-i algorithm) (Katoh and Standley 2013). Informative positions were selected by Gblocks v0.91b (default parameters, allowed gap position—“with half”) (Castresana 1997). The best-fit models of amino acid replacement were selected by ProtTest3 based on BIC scores (specified “-all-distributions” with default parameters) (Darriba et al. 2011). Finally, maximum likelihood (ML) unrooted trees were computed using RaxML v8.2.8 with branch length optimization and 1,000 rapid bootstrap replicates (Stamatakis 2006).

### Identification and Classification of GSTs in Hemipteran Species

The following procedure was applied for hemipteran species with no published classification of their GST family: interproscan v5.34-73.0 (Jones et al. 2014) was used to annotate the species transcriptome (default parameters, Pfam database v.32 and ProDom database v.2006.1) (accession numbers: *T. vaporariorum*, SRX314905; *Halyomorpha halys*, GCF_000696795.2; *Diaporina citri*, GCF_000475195.1; *Diuraphis noxia*, GCF_001186385.1; *Essigella californica*, SRX314848; *Acanthocasuarina muellerianae*, SRR921560; *Acyrthosiphon pisum*, GCA_005508785.1; *M. persicae*, GCF_001856785.1; *Paraleyrodes minei*, unpublished data, GST sequences can be found in supplementary file 1, Supplementary Material online; *Plancoccus citri*, SRX1643231; *Dialeurodes citri*, SRX1396603), and the output was scanned for sequences that were annotated with GST associated terms (IPR: 016639, 004046, 004045, 005955, 005442, 003080, 003082, 40162, 041695, 040075, 040077, 034333, 034330, and 003081 and/or PF: 00043, 02798, 13417, 13410, 16865, 14834, 14497, 13409 motives). The data retrieved were forced to include only one sequence for each transcript. Phylogenetic analysis was used to classify the sequences to the different GST classes, based on their homology with the *D. melanogaster* and *B. tabaci* data sets.

### Positive Selection Tests

Each GST gene alignment (conducted using MAFFT v7.215) included the nucleotide sequences of the six *B. tabaci* species and the sequence of the closest *T. vaporariorum* gene as an outgroup. The *B. tabaci* sequences used in each alignment were amplified by the same or similar primers and clustered as orthologous groups (supplementary fig. S2, Supplementary Material online). The outgroup, *T. vaporariorum*, was the closest branch to the *B. tabaci* orthologous groups. Similar alignments were conducted at the protein level and were later used to produce the codon alignments using PAL2NAL v14 (Suyama et al. 2006). Putative saturation of synonymous sites in the codon alignments was assessed using Xia’s test implemented in DAMBE 5 (Xia et al. 2003; Xia 2013). No relevant saturation of the phylogenetic signal was detected among all alignments (see supplementary table S4, Supplementary Material online). The protein alignments were also used to produce phylogenetic trees by RaxML v8.2.8 as detailed above (Stamatakis 2014). A “nexus” file, containing both the phylogenies and codon alignments, was then used for running the positive selection tests on each GST gene separately (codon alignments for all 25 GST genes can be found in Supplementary alignments).

The “nexus” files were uploaded to Datamonkey (https://www.datamonkey.org, last accessed January 23, 2020; Kosakovsky Pond and Frost 2005) and subjected to substitution model analyses. After determining the model that best fits each data set, Genetic Algorithm for Recombination Detection (GARD) (Kosakovsky Pond et al. 2006) tests were conducted for the detection of possible recombination events. The GARD test uses a statistical approach to identify recombination breakpoint/s in multiple-sequence alignments of homologous sequences, for inferring a unique phylogenetic history for each detected recombination block. In cases where GARD detects a significant (*P* value ≤ 0.05) breakpoint, it provides a partitioned data set that can be used for the positive selection analyses. The following site-specific positive selection tests were conducted: Mixed Effects Model of Evolution (MEME), which tests the hypothesis that individual sites have been subjected to episodic positive or diversifying selection (threshold for positively selected sites, *P* value ≤ 0.05) (Murrell et al. 2012), Fixed Effects Likelihood (FEL) which assumes that the selection pressure for each site is constant along the entire phylogeny (*P* value ≤ 0.05), Random Effects Liklihood (REL) which allows synonymous rate variation, and is often the only method that can detect selection in small (5–15 sequence) or low diverging alignments (Bayes Factor > 50) (Kosakovsky Pond and Frost 2005) and Branch-site Unrestricted Statistical Test for Episodic Diversification (BUSTED), the gene wide test, which tests if a gene has experienced positive selection in at least one site on at least one branch. BUSTED additionally calculates “evidence ratios” (ERs) for each site. The ER gives the likelihood ratio (reported on a log-scale) that the alternative model was a better fit to the data compared with the null model. The ER for each site thus provides descriptive information about whether a given site could have evolved under positive selection (choosing all *B. tabaci* branches as test branches) (Murrell et al. 2015). Codon-specific tests were considered significant if the likelihood ratio test (LRT) values of the unconstrained model likelihood scores (ln *L*_1_) versus constrained model likelihood scores (ln *L*_0_) [2 × (ln *L*_1_ – ln *L*_0_)] were ≥3.841 (i.e., *P* value ≤ 0.05 under *χ*^2^ test, DF = 1), regardless if the gene wide test gave a significant value or not. All tests were made with the user provided trees.

Additionally, two tests were run on CODEML (Yang 1997, 2007), for detecting putative episodes of positive selection: M7 versus M8 (site test) and model A (branch-site test, hereafter M2A). LRTs were performed on the likelihood scores (ln *L*) of the M7 (not allowing positively selected sites) and M8 (allowing positively selected sites) models. The M8 model was accepted when 2 × (ln *L*_1_ – ln *L*_0_) was higher or equal to 5.991 (i.e., *P* value ≤ 0.05 under *χ*^2^ test, DF = 2). In M2A, the null hypothesis assumes no positively selected sites on foreground branches (M2A_0_, ln *L*_0_), whereas the alternative hypothesis allows positively selected sites on foreground branches (M2A_A_, ln *L*_1_). The alternative hypothesis M2A_A_ was accepted when 2 × (ln *L*_1_ – ln *L*_0_) was higher or equal to 3.841 (i.e., *P* value ≤ 0.05 under *χ*^2^ test, DF = 1). For both models, the Bayes Empirical Bayes (probability ≥ 0.95) test was used to point specific sites under positive selection. The tests were applied to different phylogenetic inputs representing all possible foreground branches of a specific GST (Yang et al. 2005).

Sites were considered as positively selected if at least two tests (from Datamonkey and/or CODEML) gave significant values. In one case (position 28 in *GstD5*), the Bayes Empirical Bayes test from CODEML showed the highest posterior possibility score (0.99) for the single codon to be positively selected, whereas the LRTs of the CODEML tests were not significant. The site was considered to be positively selected because the MEME test results were also significant. In addition, we made sure that for each reported positively selected site, at least two tests indicated that the positive selection event occurred inside the *B. tabaci* clade and not only in the outgroup *T. vaporariorum*. For verification, the whole procedure was reconducted using phylogenetic trees driven from the nucleotide sequence alignments of the genes. When required, predictions of the evolutionary pathways of single sites (the most likely pathway of nucleotide substitutions) were made, using the ancestral reconstruction output of CODEML.

### Homology Modeling and the Identification of Mutations with a Potential Functional Effect

At the final stage, we looked for mutations in the vicinity of the GST enzymes’ conserved domains (the GSH binding site, the SBS and the dimer interface) by comparing the alignment of each GST to the alignments of the domains of GSTs in the CDD database (Marchler-Bauer et al. 2017). For GSTs harboring mutation/s near or in the conserved domains, a three-dimensional (3D) structure was constructed, and estimations were made for possible effect/s on the domain properties and function. These include changes in hydrophobicity, geometry, volume, and charge. Structural 3D models of the analyzed GSTs were produced using the I-TASSER web server (https://zhanglab.ccmb.med.umich.edu/I-TASSER, last accessed January 23, 2020) for structure prediction (TM-scores, *C*-scores, and Root-Mean-Square Deviations (RMSDs) are presented in supplementary table S2, Supplementary Material online) (Yang et al. 2015). Alignments of structural models of isozymes and visualization of the models were done using UCFC Chimera v1.13.1 (Pettersen et al. 2004).

## Results

### Classification of the *B. tabaci* GST Family and Its Relation to Other Hemipteran Species

**Fig. 1.**
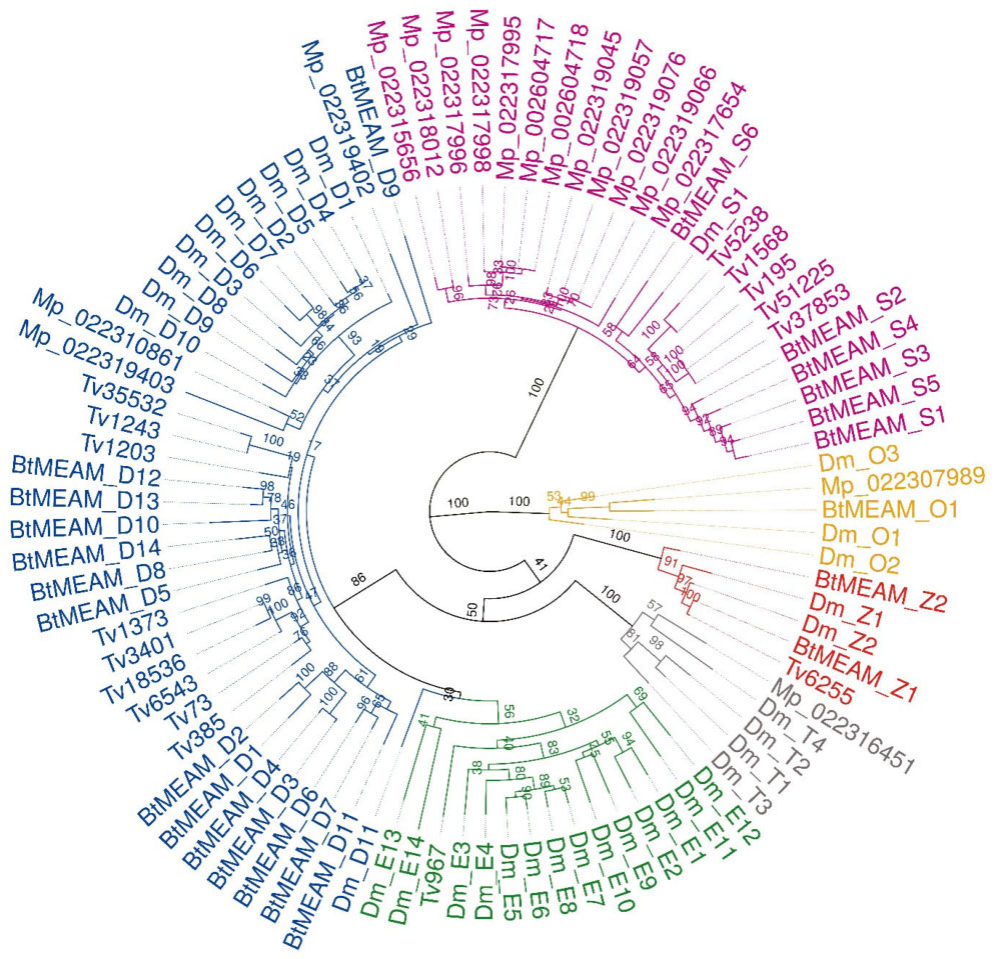
—ML tree of hemipteran insects and *Drosophila melanogaster* cytosolic GST proteins. Clades of classes: blue, Delta (D); green, Epsilon (E); gray, Theta (T); orange, Omega (O); red, Zeta (Z); purple, Sigma (S). Dm, *Drosophila melanogaster*; BtMEAM, *Bemisia tabaci* MEAM1; Mp, *Myzus persicae*; Tv, *Trialeurodes vaporariorum*. Bootstrap support values (1,000 replicates) are displayed at each node.

In order to classify the *B. tabaci* GST gene family into its different classes, we produced a phylogenetic tree that contains GST sequences from *B. tabaci*, *D. melanogaster*, *M. persicae*, and *T. vaporariorum* (fig. 1). This resulted in well separated clades for each class. *Bemisia* *tabaci* appears to have four classes of cytosolic GSTs: 14 genes in the Delta class, 1 gene in the Omega class, 6 genes in the Sigma class, and 2 genes in the Zeta class. In addition, *B. tabaci* harbors two microsomal genes. Genes belonging to the Epsilon or Theta classes were not found. The obtained classification of the *B. tabaci* cytosolic GSTs was consistent with the automatic annotations made by the MEAM1 genome project (Chen et al. 2016), with one exception. Our work identified the presence of 25 GST genes in *B. tabaci* (table 1), which is one more than what was discovered in the MEAM1 genome project (Chen et al. 2016). The additional *GST* was not detected previously, because its sequence was split between two scaffolds. We used the nomenclature proposed by Chen et al. (2016) and named the additional gene *GstS6* (fig. 1). In addition, our GST gene list of the MED (Mediterranean) species, is markedly different from the one published by the MED genome project, which reported the identification of 21 GST genes (Xie et al. 2018). Contrarily, our PCR amplification and sequencing analysis recovered 24 GSTs in MED-Q2. The only gene that could not be amplified in MED-Q2 was *GstD3*, likely due to its high sequence similarity to *GstD4* (94% identity at the nucleotide sequence level in MEAM1), which made it impossible to differentiate between the two genes by primer-specific PCR amplification (from cDNA samples) or assembly of transcriptomes (reads from the two genes could not be uniquely mapped). However, we did find most of the *GstD3* sequence in the published MED genome (Xie et al. 2018) in vicinity to the *GstD4* locus, suggesting synteny between the MED and MEAM1 genomes. *GstD3* was also found to be present in NW2, ASIAII-1 (PCR amplifications), and SSA1-SG3 (unpublished genomic data). Therefore, it can be carefully argued that at least 4 of the 6 *B. tabaci* species analyzed in this study share a common set of 25 GST genes (with the current sequencing data, we could not determine if *GstD3* is present in Australia), and we also could not amplify *GstD10* from ASIAII-1 cDNA or detect it in the transcriptomic data of the species (the GST phylogenetic tree that includes all 6 analyzed *B. tabaci* species is presented in supplementary fig. S2, Supplementary Material online). This seems to be one of the largest sets of GST genes described so far in any hemipteran species (table 1).

**Table 1:** Numbers of GST Genes in Hemipteran Insects

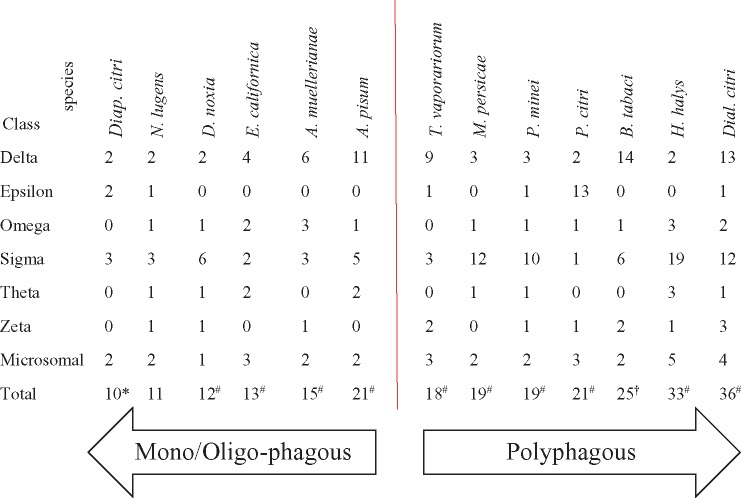

Note.—*Diap. citri*, *Diaphorina citri* (Psylloidea) (GST numbers and host range by Arp et al. [2016] and *Diaphorina citri* (Asian citrus psyllid) [2019], respectively); *N. lugens*, *Nilaparvata lugens* (Delphacidae) (GST numbers and host range by Xue et al. [2014] and Yuan et al. [2005], respectively); *D. noxia*, *Diuraphis noxia* (Aphididae) (host range by Botha [2013]); *E. californica*, *Essigella californica* (Aphididae) (host range by Wharton and Kriticos [2004]); *A. muellerianae*, *Acanthocasuarina muellerianae* (Psylloidea) (host range by Taylor et al. [2011]); *A. pisum*, *Acyrthosiphon pisum* (Aphididae) (host range by Sandstrm [1994]); *T. vaporariorum*, *Trialeurodes vaporariorum* (Aleyrodidae) (host range by Poprawski et al. [2000]); *M. persicae*, *Myzus persicae* (Aphididae) (host range by Ali and Agrawal [2012]); *P. minei*, *Paraleyrodes minei* (Aleyrodidae) (host range by Mound and Halsey [1978]); *P. citri*, *Planococcus citri* (Coccoidea) (host range by Ahmed and Abd-Rabou [2010]); *B. tabaci*, *Bemisia tabaci* (Aleyrodidae) (host range by Malka et al. [2018]); *H. halys*, *Halyomorpha halys* (Pentatomidae) (host range by Leskey and Nielsen [2018]); *Dial. citri*, *Dialeurodes citri* (Aleyrodidae) (host range by Mound and Halsey [1978]).

^a^ *Diaphorina citri* has one uncharacterized GST.

^b^Numbers are based on our own new analysis of the published transcriptome (see Identification and Classification of GSTs in Hemipteran Species).

^c^The possibility for some variation in numbers among the *B. tabaci* species could not be ruled out.

**Fig. 2.**
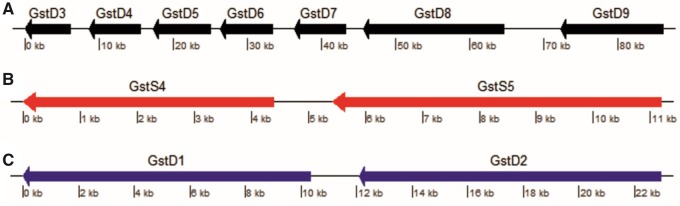
—Clusters of GST genes in the MEAM1 genome. Coordinates were taken from the first version of the whitefly genome (Chen et al. 2016), (*A*) scaffold 147: 2852369–2938330, (*B*) scaffold 1034: 4126943–4139168, and (*C*) scaffold 533: 851824–863005.

Only two one-to-one orthologs GSTs were found to be shared between *B. tabaci* and the other analyzed species: *D. melanogaster* *GstS1* and *B. tabaci* *GstS6* and *T. vaporariorum* Contig 6255 and *B. tabaci* *GstZ1*. No one-to-one orthologous GSTs were found between *B. tabaci* and *M. persicae*, which might indicate an independent expansion of the GST family in each species (table 1 and fig. 1). The Delta class, known to have a role in detoxification of xenobiotics (Ranson and Hemingway 2005; Friedman 2011; Shou-min 2012), seems to be expanded (14) in all analyzed *B. tabaci* species (table 1). Examination of the chromosomal organization and orientation of the 25 GST genes in the *B. tabaci* genome (MEAM1) identified three clusters (defined here as a group of genes with a maximal distance of 10,000 base pairs between one gene and its neighbor): *GstD3*-*GstD9*, *GstS4*-*GstS5*, and *GstD1*-*GstD2* (fig. 2). Our phylogenetic analyses suggested, however, that duplications might have occurred only in three pairs: *GstD1* and *GstD2*, *GstD3* and *GstD4*, and *GstD6* and *GstD7*, which clustered as the closest phylogenetically related gene one to each other (fig. 1).

Next, we searched for expansion (size) patterns of the GST family among different hemipteran (mostly sternorrhynchan) species with different documented host range (see table 1 for details). *Diap. citri* (psyllid), *N. lugens* (planthopper), *D. noxia* (aphid), *E. californica* (aphid), and *A. muellerianae* (psyllid), which specialize on citrus, rice, cereal grasses, pines, and slaty sheoak, respectively, displayed considerably reduced numbers of GST genes in their genomes compared with *T. vaporariorum* (whitefly), *M. persicae* (aphid), *P. minei* (whitefly), *P. citri* (mealybug), *B. tabaci* (whitefly), *H. halys* (stink bug), and *Dial. citri* (whitefly), all known to be capable of feeding on a large number of plant hosts (table 1). The only exception was *A. pisum* (aphid). Our analysis indicated that the genome of *A. pisum* contains 21 GST genes although the species feeds solely on Fabaceae plants.

### Positive Selection Analyses

**Fig. 3.**
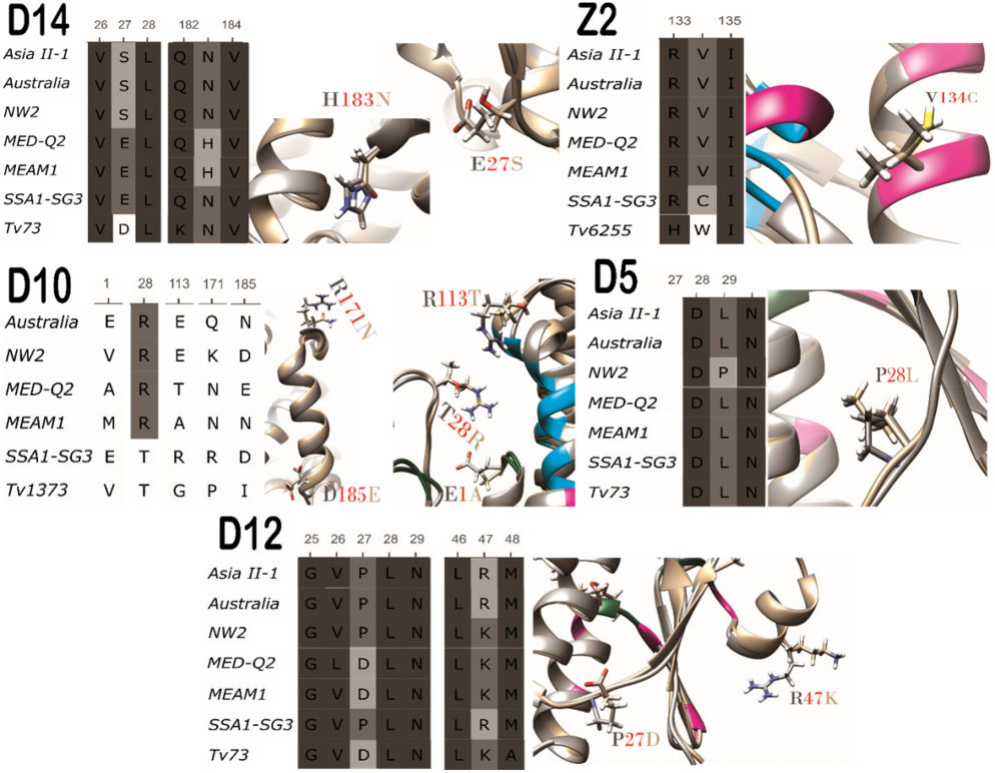
—Positively selected residues in cytosolic GSTs. Amino acids sequence alignment (left). Protein structure alignment 3D image (right). Off-white and gray stands for the different variants. The positively selected residues are tagged. Magenta backbone stands for dimer interface residues. Light blue for substrate binding residues. Green for glutathione binding residues. A positively selected residue (132) found in GstMicrosomal2 is not shown due to lack of sufficient data on its 3D structure and function. TM-scores, *C*-scores, and RMSDs of the models are presented in supplementary table 3, Supplementary Material online.

Positive selection was found to act on residues of 6 (GstD14, GstD12, GstD10, GstD5, GstZ2, and GstMicrosomal1) out of the 25 GSTs of *B. tabaci* (indication of significance in at least 2 tests) (fig. 3 and supplementary table 3, Supplementary Material online). In total, 12 amino acid residues were found to evolve under positive selection, 10 of them in GST enzymes belonging to the Delta class, 1 in an enzyme from the Zeta class, and 1 in a microsomal enzyme. The GARD test found significant evidence for recombination in 2 of the Delta class genes, in codon 124 of *GstD12* (*P *=* *0.025) and in codon 66 of *GstD14* (*P *=* *0.01). In *GstD12*, the location of the two positively selected residues is before the breakpoint (codons 27 and 47), whereas in *GstD14*, the positive selected residue (codon 183) is located after the breakpoint. In both cases, the positive selection analyses utilized partitioned data sets, where different sites were allowed to evolve according to different phylogenies (i.e., a recombination-corrected data set from GARD).

**Fig. 4.**
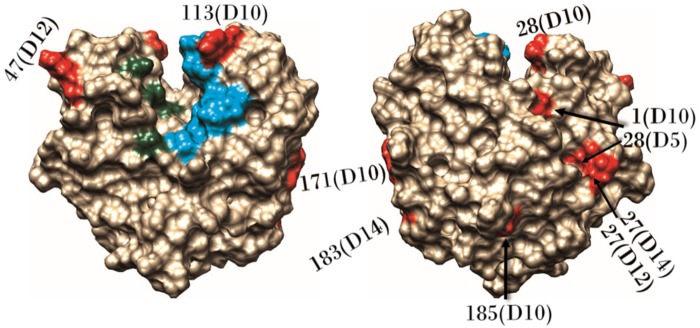
—Location of positively selected sites in Delta GSTs. Positively selected sites (red) were detected in the periphery of Delta GSTs. The location of each residue and the GST in which the mutation occurred is presented (gene name includes class and number only). Light blue backbone stands for substrate binding residues, green for glutathione binding residues, as marked in the NCBI Conserved Domain Database. Left, a view from the inner part of the dimer on the interior side of one of the subunits. Right, a view from the outer part of the subunit.

Ten of the 11 positively selected residues in cytosolic GSTs were found to be located in the periphery of the GST dimer (fig. 4). The 11th residue was found to be oriented toward the interface between the two subunits of the dimer (GstZ2—V134C, not shown in fig. 4). One of the peripheral mutations mentioned above was found in the substrate entrance site to the catalytic cavity (GstD10—R113T). Our analyses did not detect any positively selected sites that are located in the hydrophobic core of the subunits (fig. 4). Three positively selected sites were found in a single loop, between α1 and β2 (supplementary fig. S1, Supplementary Material online) of three different GSTs (position 27 in GstD12 and GstD14 and position 28 in GstD5). The two mutations in position 27 seem to affect the charge of the residue. In GstD12, the mutation is also expected to reduce the flexibility of the backbone (fig. 3).

### The Evolutionary Pathway of Positively Selected Sites

Of the 12 positively selected substitutions, 5 were found to be present only in 1 species or to be shared between closely related species from the same clade (residues: 134 in GstZ2, 183 in GstD14, 28 in GstD5, 28 in GstD10, and 132 in GstMicrosomal2). These substitutions likely occurred once in the specific species or in the common ancestor of the clade. Four more positively selected substitutions were found in GstD10 (residues 1, 185, 117, and 113). These substitutions occurred in multiple species in each residue. *GstD10* can be considered, therefore, as a fast evolving gene (also reflected by its long branch in fig. 1), or alternatively, a gene under a pseudogenization process. The remaining three positively selected residues (position 27 in GstD14 and GstD12 and position 47 of GstD12) showed a more complicated evolutionary course that could not be simply explained by the species relative phylogenetic relatedness.

**Fig. 5.**
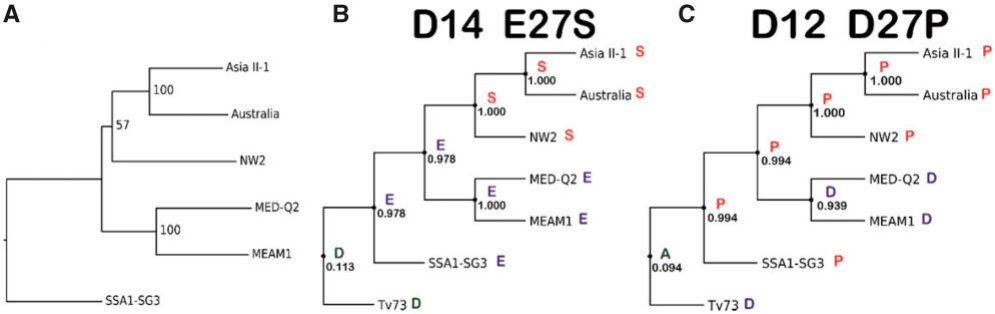
—Substitutions evolutionary pathway. The ancestral reconstruction of positively selected residues by CODEML. Amino acids in external nodes are validated by sequencing. Amino acids in internal nodes are predicted by reconstructing the most likely ancestral residue in each position. The relevant posterior probability is presented underneath each of the amino acids of the internal nodes. (*A*) ML phylogenetic tree that was generated based on the concatenated data of all 25 GST sequences (bootstrap support values are displayed at each node representing 1,000 replicates). (*B*) Ancestral sequence reconstruction of position 27 in GstD14. (*C*) Ancestral sequence reconstruction of position 27 in GstD12.

We tracked the putative ancestral sequence and the most plausible evolutionary pathway of each of the three aforementioned residues using the ancestral sequence reconstruction method in CODEML, using the concatenated GST family tree as a guide (fig. 5*A*). The tree indicated that the SSA1-SG3 species was the first to diverge. The next major split occurred between the Asian and the Mediterranean/Asia Minor/Africa lineages. The NW2 species was found to be phylogenetically related to the Asian clade of *B. tabaci*, as previously suggested by other phylogenetic studies that utilized nuclear gene alignments instead of the commonly used *mtCOI* marker (Hsieh et al. 2014). According to the ancestral sequence reconstruction of position 27 in GstD14, only one amino acid substitution, from glutamic acid to serine in the common ancestor of NW2, Asia II-1, and Australia, is required to establish a highly likely (supported by high posteriors) evolutionary pathway (fig. 5*B*). In position 27 of GstD12, the common ancestor of the *B. tabaci* species complex likely harbored proline, a noncharged amino acid residue, and the substitution occurred in the common ancestor of the MEAM1 and MED-Q2 species, changing the residue to an acidic one (aspartic acid) (fig. 5*C*). Interestingly, the presence of an acidic residue at position 27 was found to be conserved among other Delta class GST enzymes of *B. tabaci* (data not shown). A well-supported reconstruction pathway for position 47 in GstD12 could not be produced.

### Functional Changes Associated with Amino Acid Substitutions in the GST Family of *B. tabaci*

**Fig. 6.**
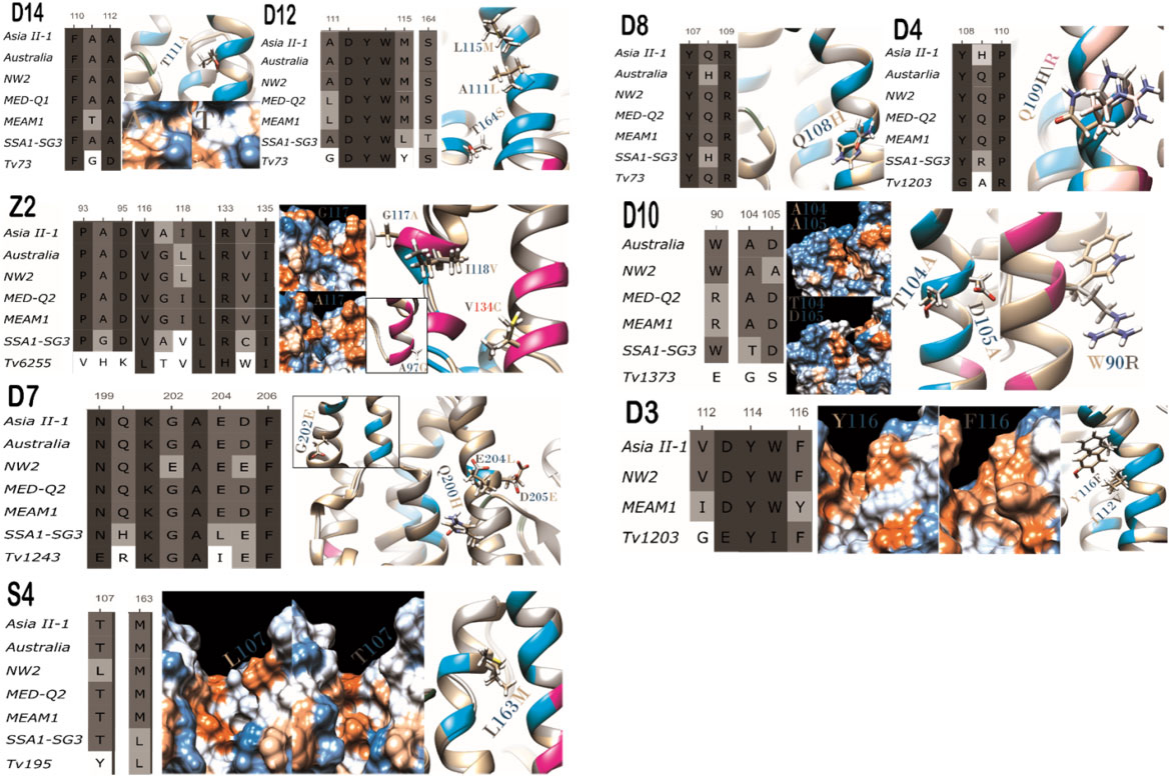
—Mutations that have the potential to affect the substrate specificity and/or enzyme activity. From left to right: amino acid sequence alignment followed by a 3D image. The substituting residue structures and tags are presented in off-white, gray, and light-pink. Pink backbone indicates that the residue is located in the dimer interface, light blue indicates a residue putatively involved in substrate binding, and green indicates a residue putatively involved in glutathione binding. For GSTs D14, Z2, S4, D10, and D3, hydrophobicity surface maps of the two variants are also presented. Red indicates a hydrophobic residue, white indicates a neutral residue, and blue indicates a hydrophilic residue. TM-scores, *C*-scores, and RMSDs of the models are presented in supplementary table 3, Supplementary Material online.

In addition to the detection of positively selected sites, we searched the GST family of *B. tabaci* for the presence of mutations that could have an effect on the function/s of the enzymes. We considered only mutations that occurred in the vicinity of the catalytic cavity or the dimer interface, and therefore have the potential to change the enzyme’s chemical characteristics or structure (i.e., could cause different affinity to the enzyme’s substrates or differences in the substrates identity). In total, 21 mutations of the 2 types were identified, including 2 (GstZ2—V134C, GstD10—R113T) that also showed a positive selection signature. The 21 mutations were classified into 4 categories that specify their potential impact on the properties of the enzymes (some mutations were classified to more than 1 category): 1) Mutations found in GstD12 (L115M, T164S), GstD14 (T111A), GstZ2 (G117A), GstS4 (L163M, T107M), GstD10 (T104A, D105A, E113T/A), and GstD3 (Y116F), which alternate between residues with different hydrophobicity characters. These mutations are expected to change the hydrophobic characters of the surface of the SBS (figs. 3 and 6). 2) Mutations found in GstZ2 (V134C, G117A, I118A, A94G) and GstD10 (W90R) are located in the dimer interface. As a result, they can alter the shape and volume of the active cavity, by changing the geometry of the interactions between the two subunits (figs. 3 and 6). For example, the change from glycine to alanine in residue 117 of GstZ2. Glycine has a single hydrogen atom at its side chain allowing extra flexibility of the backbone compared with alanine. When taking into account also the variation in the amino acids in the proximate position 118, it is highly likely that the structure of the backbone changes between the two variants. 3) Mutations found in GstD12 (L115M, A112L), GstS4 (L163M), and GstD10 (T104A, D105A) reflect changes in the volume of the amino acids, which likely affect the active cavity shape and volume and therefore the catalytic activity of the enzymes (fig. 6). 4) Mutations in positions 116 and 112 of GstD3, 108 of GstD8, 109 of GstD4, and 111 of GstD14 likely effect substrate binding, as they are expected to allow the establishment of different bonds and/or orientations between the enzymes and the substrates and therefore to affect the affinity between them. For example, the change from glutamine to histidine (positions 108 in GstD8 and 109 of GstD4) changes the orientation of a possible hydrogen bonding with the substrate and adds the possibility to create Phi bonds and salt bonds when charged (p*K*_a_ = 6.04). The Arginine in GstD4 (position 109) is larger than the alternative residues and is also positively charged constantly. The change from phenylalanine to tyrosine in GstD3, position 116, allows to establish hydrogen bonds with the substrate and occurs together with the V112I mutation which is also located in the SBS. T111A in GstD14 also makes it possible to establish hydrogen bonds with the substrate (fig. 6).

## Discussion

### The Size and Composition of the *B. tabaci* GST Family

All 25 genes of the *B. tabaci* GST family were found to be present in the representative of the SSA genetic group (SSA1-SG3), the first group to diverge within the *B. tabaci* species complex. Also, this full-set was found in at least one species of the two main derived lineages, the Africa/Middle East/Asia Minor and Asian clades (Boykin et al. 2013). This likely indicates that the common ancestor of the species complex also had a similar set of 25 genes. The *B. tabaci* GST gene set is among the largest described so far among the Hemiptera (table 1). Most of the expansion occurred in the Delta class, which is unique to insects and was previously shown to be involved in the detoxification of xenobiotics (Chen et al. 2003; Shou-min 2012)*.* Our finding that nearly no one-to-one ortholog GSTs are shared between *B. tabaci* and *M. persicae* (aphid) or *T. vaporariorum* (whitefly), suggests that the expansion of the GST family and specifically the Delta class in *B. tabaci* occurred independently. Nevertheless, we cannot rule out the possibility that the evolution of some common ancestral GSTs in hemipterans took place under a relaxed selection pressure or under a strong selection pressure. This is expected to lead, in each group, to the accumulation of significant changes in the genes’ sequences (and functions), making the detection of orthologous genes impossible.

Our data raised an interesting possibility that an association exists between the diet breadth of the hemipteran species and their total number of GST genes (table 1). As indicated earlier in the manuscript, this idea is not new as clear relationship between the numbers of detoxification genes and host use was found in the hemipteran species analyzed by Rane et al. (2016, 2019). Different from our findings, the variation reported by Rane et al. (2016) was mostly associated with differences in the numbers of P450 and COE genes and not with a variation in the size of the GST families. With caution, due to the relatively little genomic/transcriptomic data available today (we analyzed here 13 species), it can be stated that it is a necessary but insufficient prerequisite for polyphagous hemipteran species to harbor at least ∼20 GST genes, and to show a prominent expansion in the Delta/Epsilon and/or Sigma classes. These two classes are known to be involved in the neutralization of plant secondary metabolites and xenobiotics and are considered to be environmental response genes (Singh et al. 2001; Sawicki et al. 2003). Expansions in each family size could therefore lead to improved and/or more sophisticated ability to handle various toxic challenges in multiple environments (Ranson and Hemingway 2005; Shou-min 2012). One exception in table 1 is *A. pisum*, which feeds solely on Fabaceae plants (Sandstrm 1994) and has one more GST gene than the polyphagous species *M. persicae* (table 1). In this case, it is more likely that the diet breadth differences between these two aphids species are at least partially associated with the significant expansion of the P450 gene family in *M. persicae* (Simon et al. 2015). Expansion of the P450 gene family might also explain the reported wide host range of multiple species within the *B. tabaci* species complex (Malka et al. 2018). Recent studies already documented an expansion of the detoxifying CYP3 and CYP4 gene clades in the MED and MEAM1 species of *B. tabaci*, when compared with other sap-feeding, blood-feeding, and other insect groups (Xie et al. 2018), bringing additional support for the possible linkage between detoxification activity/ability, ability to feed on a broad host range and insecticide resistance traits present in some species within the *B. tabaci* complex. Nevertheless, the findings of this research and those of a parallel work conducted on the P450s family (Juravel 2018) bring evidence that there is no size differences in the GST or P450 gene families among species within the *B. tabaci* complex. Therefore, the reported variations in host range within the complex likely result from different expression patterns of the detoxification genes (Malka et al. 2018) and/or differences in the coding sequence of these genes (see more details below).

### Positive Selection in *B. tabaci* GSTs and Their Putative Function

The identification of a positive selection signature highlights mutations that get fixed in the population due to the advantage they give to individuals carrying them (Yang et al. 2000; Yang 2006). In this work, we found positive selection to act on residues of five Delta GSTs in *B. tabaci*. Initially, our working hypothesis was that positively selected residues will be found at the enzymes’ sites involved in determining their efficiency, affinity to substrate/s, and substrate range. This working hypothesis was supported by previous studies, which focused on changes in amino acid residues that are located in or near the active pocket of the studied GST enzyme/s (Ivarsson et al. 2003; Wai et al. 2007; Matzkin 2008; Da Fonseca et al. 2010; Lan et al. 2013). For example, Matzkin (2008) studied the GstD1 enzyme of the cactophilic fly *Drosophila mojavensis* and found that positive selection acting on specific amino acids within the active site pocket of the GSTD1 enzyme was likely to be involved in adaptation to columnar cactus hosts in the Baja/Sonora lineage of *Drosophila* *mojavensis*. Further support was provided later by biochemical analysis which revealed functional differences between the GstD1 isoforms (Matzkin 2014). However, 10 of the 11 positively selected residues identified in this study were found to be located in the periphery of the GST dimer, making it difficult to outline the putative mechanism that enables these mutations to be advantageous to their hosts. One possibility is that the peripheral region functions as an interface for protein–protein interactions (Qian and Zhang 2014). For example, Delta class GST enzymes from *D. melanogaster* were found to interact with a p38b mitogen-activated protein kinase, involved in cellular stress responses (Wongtrakul et al. 2012). Using interaction in vitro assays, the study demonstrated that the presence of two GSTs (DmGSTD8 and DmGSTD11b) significantly increased the kinase activity of p38b toward two transcription factors ATF2 and JUN, and the presence of two additional GSTs (DmGSTD3 and DmGSTD5) increased the kinase activity of p38b only toward JUN. In addition, the reverse effect also occurred. The activity of some Delta GSTs toward the standard artificial substrates, 1-chloro-2,4-dinitrobenzene and 1,2-dichloro-4-nitrobenzene, significantly changed in the presence of p38b (both increased and reduced activities were observed) (Wongtrakul et al. 2012). In another study, a computational two-hybrid-based protein interaction map of the *D. melanogaster* proteome identified five Delta, one Epsilon, and one Sigma GSTs that interact with other proteins including the subunit beta of an ATP synthase (Giot et al. 2003). Interestingly, three of the positive selection events we identified were found to be located in a single loop between α1 and β2 of the relevant GST enzymes (supplementary fig. S1, Supplementary Material online), highlighting this region as a possible hot spot for advantageous mutations, putatively playing an important role in protein–protein interactions.

### Putative Links between Amino Acid Variation in GST Enzymes and the Species’ Biology

In a previous work (Malka et al. 2018), we identified two GST genes in *B. tabaci* that show plastic expression in more than one species in response to host plant shifts. In addition, 13 genes were shown to be significantly and constitutively over- or under-expressed in one of the species compared with all others (five of the species previously analyzed were also included in this study). From them, four genes are relevant also to this study, the genes coding for GstD5, GstS4, GstD12, and GstZ2. In our previous study, *GstD5* was found to be significantly downregulated in the NW2 species which was also found to be more “restricted” in its host range compared with other species in the complex. Here, we found that GstD5 in NW2 contains a positive selection site at position 28 (L28P), located in the periphery of the GST dimer. The proline at this position is expected to add extra rigidity to the structure that might interfere with the enzyme’s ability to be involved in protein–protein interactions (Qian and Zhang 2014). Taking together the two finings suggests reduced functionality of GstD5 in NW2, which might be related to a more general process of reduced/changed involvement of the protein in detoxification as part of a specialization process on a narrow range of host plants (Ragland et al. 2015). Similar interpretation can be applied to the T107L change in GstS4 of NW2, which is expected to change the hydrophobic characters of the surface of the SBS (fig. 6), and the functionality of the enzyme relative to the GstS4 enzymes of other species in the complex. Interestingly, GstS4 was found to be constitutively and plastically overexpressed in three species of *B. tabaci* that were analyzed here that can be considered as generalist species with “extended” host ranges (SSA1-SG3, ASIAII-1, and MED-Q1) (Malka et al. 2018), suggesting some involvement of the GstS4 enzyme in successful host adaptation. In addition, we show here that the enzymes GstZ2 and GstD12 harbor three positive selection sites (residues: 134 in GstZ2 and 27, 47 in GstD12) and a total of seven mutations that can have an effect on the function/s of the enzymes (residues: 94, 117, 118, 134 in GstZ2 and 111, 115, 164 in GstD12). Most of these mutations appear solely in SSA1-SG3 (five) and therefore can also be associated with random genetic drift (see below) as the SSA1-SG3 species was the first to diverge (fig. 5*A*). However, two of the positively selected changes (D27P and R47K in GstD12) and one of the function changing mutations (G117A) are present in both SSA1-SG3 and ASIAII-1, two *B. tabaci* species with “extended” host range but nonoverlapping geographic distribution (De Barro et al. 2011). Moreover, as these two species appear in two different clades of the *B. tabaci* phylogeny (Hu et al. 2014), it might be assumed that they have retained the amino acid residues that were already present in the common ancestor of the *B. tabaci* species complex (shown here for D27P in GstD12), which likely displayed a relatively “extended” feeding habit (Malka et al. 2018). As we indicated previously, the change from glycine to alanine in residue 117 of GstZ2 can alter the shape and volume of the active cavity, as it is located in the dimer interface. The gene coding for the GstD12 enzyme was previously shown to be overexpressed both in the ASIAII-1 and SSA1-SG3 species relative to all other analyzed species. In addition, the gene coding for GstZ2 was uniquely overexpressed in ASIAII-1. Taken together, these findings suggest a putative role to the ASIAII-1 and SSA1-SG3 variants of the two enzymes in the ability of the two species to feed on an “extended” range of host plants.

### Duplications of GSTs Genes in *B. tabaci* Might Allow Neofunctionalization

Acquisition of new protein–protein interaction abilities is also linked to “neofunctionalization” events (Qian and Zhang 2014). According to the “classical model,” “neofunctionalization” is the process in which, after gene duplication, one copy of the coded enzyme is free to evolve a new function, whereas the other preserves the ancestral enzyme function (Prince and Pickett, 2002; Taylor and Raes 2004). Our phylogenetic data (fig. 1) supported three duplication events involving six GST genes. This might be a conservative estimate as the chromosomal organization of the GST family in the MEAM genome, raised the possibility that expansion by duplication (Lynch and Conery 2000) involved nine genes from the Delta class and two genes from the Sigma class, located within three gene clusters (fig. 2). Moreover, from the seven GSTs (GstS4, GstD3, GstD4, GstD8, GstD10, GstD12, and GstD14) that were found to harbor mutations that likely affect their catalytic activity and function, four (GstS4, GstD3, GstD4, and GstD8) are part of these gene clusters. In general, it is predicted that duplicate genes that have been stably maintained in the genome for millions of years, mostly take an evolutionary route in which “neofunctionalization” that provides fitness benefits occurs relatively late. It is preceded by a step in which “subfunctionalization” by complementary degenerative mutations occurs in both gene copies making it necessary for the organism to selectively maintain both gene copies in the genome (He and Zhang 2005; van Hoof 2005; Hittinger and Carroll 2007; Innan and Kondrashov 2010; Teufel et al. 2019). To test this scenario, the simultaneous deletion of a duplicate gene pair in *Schizosaccharomyces cerevisiae* was compared with the deletion of their singleton counterpart in *Schizosaccharomyces pombe*. The deletion of the two copies of duplicated genes in *Schizosaccharomyces* *cerevisiae* was found to cause a greater loss of fitness than the deletion of the one copy homologous gene in *Schizosaccharomyces* *pombe*, providing unambiguous evidence that “subfunctionalization” can be followed by “neofunctionalization” for improving the organism fitness (He and Zhang 2005; Qian and Zhang 2014). Therefore, our work is only the tip of the iceberg in understanding the functionality of the GST family expansion in *B. tabaci*. Future functional and silencing analyses are required to identify the specific contribution of each gene, especially in the expended Delta and Sigma classes, to the insect’s fitness, and the level of complementation between the paralogous groups.

In summary, this work provides an overview of the evolutionary changes that occurred in the coding sequence of the GST family (all 25 genes) in the *B. tabaci* species complex. The analysis of 6 species, representing 5 of the 6 major genetic clades, identified 12 positive selection events and 21 potentially function-altering mutations. It also revealed that each analyzed species harbors a slightly different GST tool box. As most of the mutations were found to be present in the environmentally responsive classes of the GST family, Delta and Sigma, we carefully speculated (see above) that at least some of the reported changes might improve the fitness of the species carrying them, leading to a possible better adaptation of some species to specific environments. However, we are aware that drift and natural selection do not generally act in isolation, and their effect on the genome is largely dependent on population size (Bernardo et al. 2019). We have previously raised the possibility that the ancestral ability of *B. tabaci* to perform well on multiple hosts might have played a passive role in the evolution of the complex, by enhancing the probability for geographical separation between populations, leading eventually to allopatric speciation (Malka et al. 2018). Therefore, we cannot ignore the possibility that population size reduction due to migration followed by fragmentation and isolation could have also led to an increase in the frequency of effectively neutral nonsynonymous variants, brining eventually to the stochastic fixation of mutations in GST genes among different species in the complex (Bernardo et al. 2019).

## Data Availability

All alignments used are available at https://doi.org/10.6084/m9.figshare.8937992.

## Supplementary Material

evaa002_Supplementary_Data

## Notes

**Data deposition:** This project has been deposited at NCBI BioProject database under the accession PRJNA554492.
