# Supplementary material for: Molecular Evolution of the Glutathione S-Transferase Family in the Bemisia tabaci Species Complex
Source: Genome Biol Evol. 2020 Jan 23;12(2):3857–72. doi: 10.1093/gbe/evaa002 (PMC7058157; doi:10.1093/gbe/evaa002)

**Supplemental Fig. S1. – The 3-dimensional (3D) structure of a Delta GST dimer, showing the active sites and pockets.** Visualization was achieved using the X-Ray diffraction-solved-structure of the *Musca domestica* Delta class GST (Sue & Yajima 2018). Magenta backbone stands for dimer interface residues, blue for substrate binding residues, green for glutathione binding residues, as marked in the NCBI [Conserved Domain Database](https://www.ncbi.nlm.nih.gov/cdd/). **A.** Side view of GST Delta class dimer 4th structure. Red arrow demonstrate the possible entrance route of substrates to the active pocket and also stands for the angle from which C is presented. Blue arrow stands for the angle from which B is presented. **B.** Monomer GST with alpha-helix and beta-strands as described in Wang at al. (2008). **C.** Hydrophobicity surface demonstration of the dimer active pocket – red stands for hydrophobic residues, white for neutral residues and blue for hydrophilic residues. The bound glutathione is represented by sticks. **D.** Zoom in on the SBS residues. E. Zoom in on the glutathione binding residues, the bound Glutathione is represented by orange sticks.

Sue M, Yajima S. 2018. Crystal structure of the delta-class glutathione transferase in *Musca domestica*. Biochem Bioph Res Co. 502:345-350.

Wang Y et al. 2008. Structure of an insect epsilon class glutathione S-transferase from the malaria vector *Anopheles gambiae* provides an explanation for the high DDT-detoxifying activity. J Struct Biol. 164:228-235.


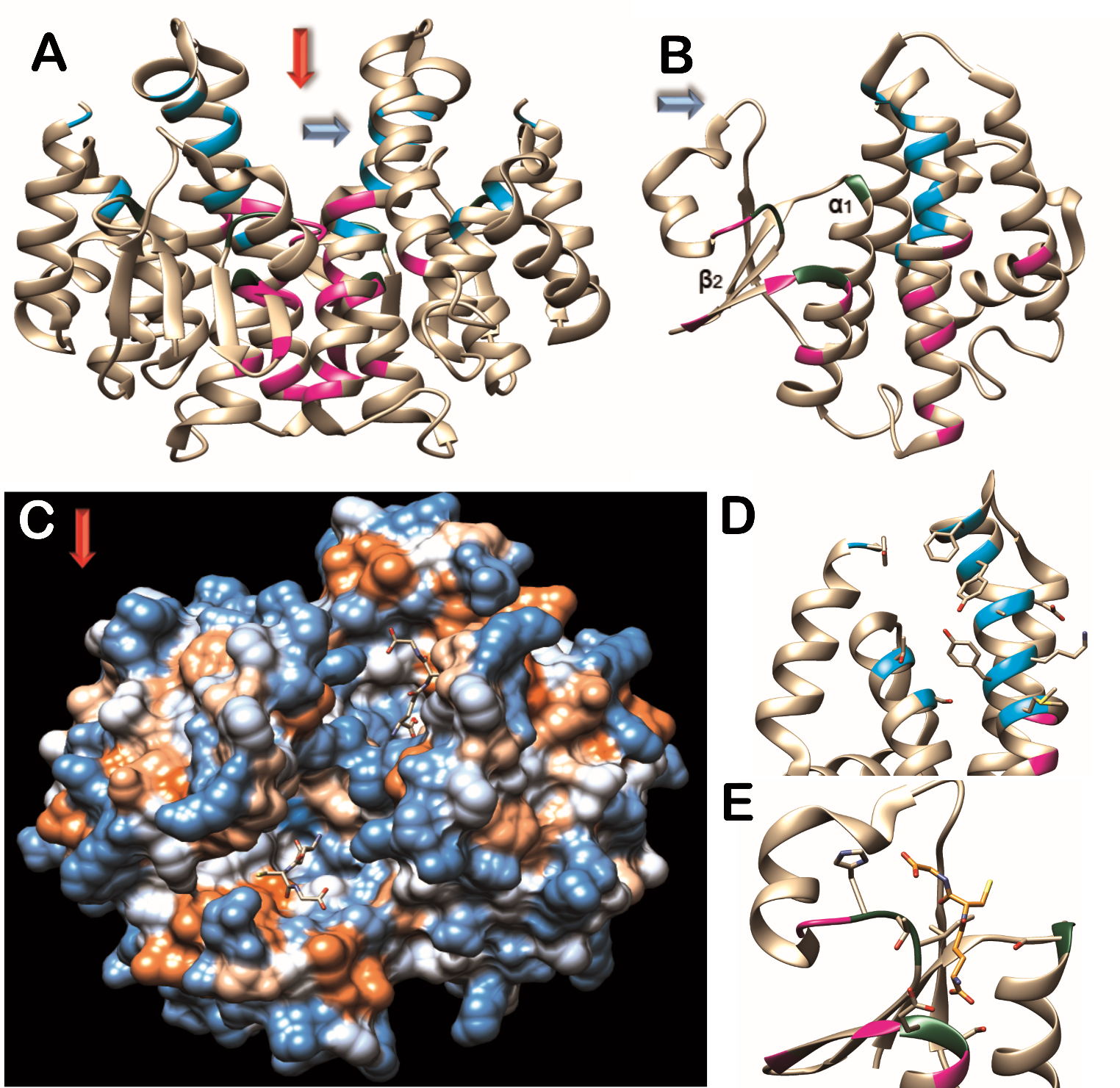

Supplement: evaa002_Supplementary_Data [file evaa002_supplementary_data.zip › Supplementary figure S1 revised version.docx]
