# Supplementary material for: Molecular Evolution of the Glutathione S-Transferase Family in the Bemisia tabaci Species Complex
Source: Genome Biol Evol. 2020 Jan 23;12(2):3857–72. doi: 10.1093/gbe/evaa002 (PMC7058157; doi:10.1093/gbe/evaa002)

**Supplemental Fig. S2. – Maximum likelihood tree of cytosolic GST proteins from all six analyzed *B. tabaci* species and *D. melanogaster*.**

Clades of classes: purple – Delta (D), Green – Epsilon (E), Grey – Theta (T), Orange – Omega (O), Red – Zeta (Z), Pink – Sigma (S). Dm - *D. melanogaster*. Bootstrap support values (1000 replicates) are displayed at each node.


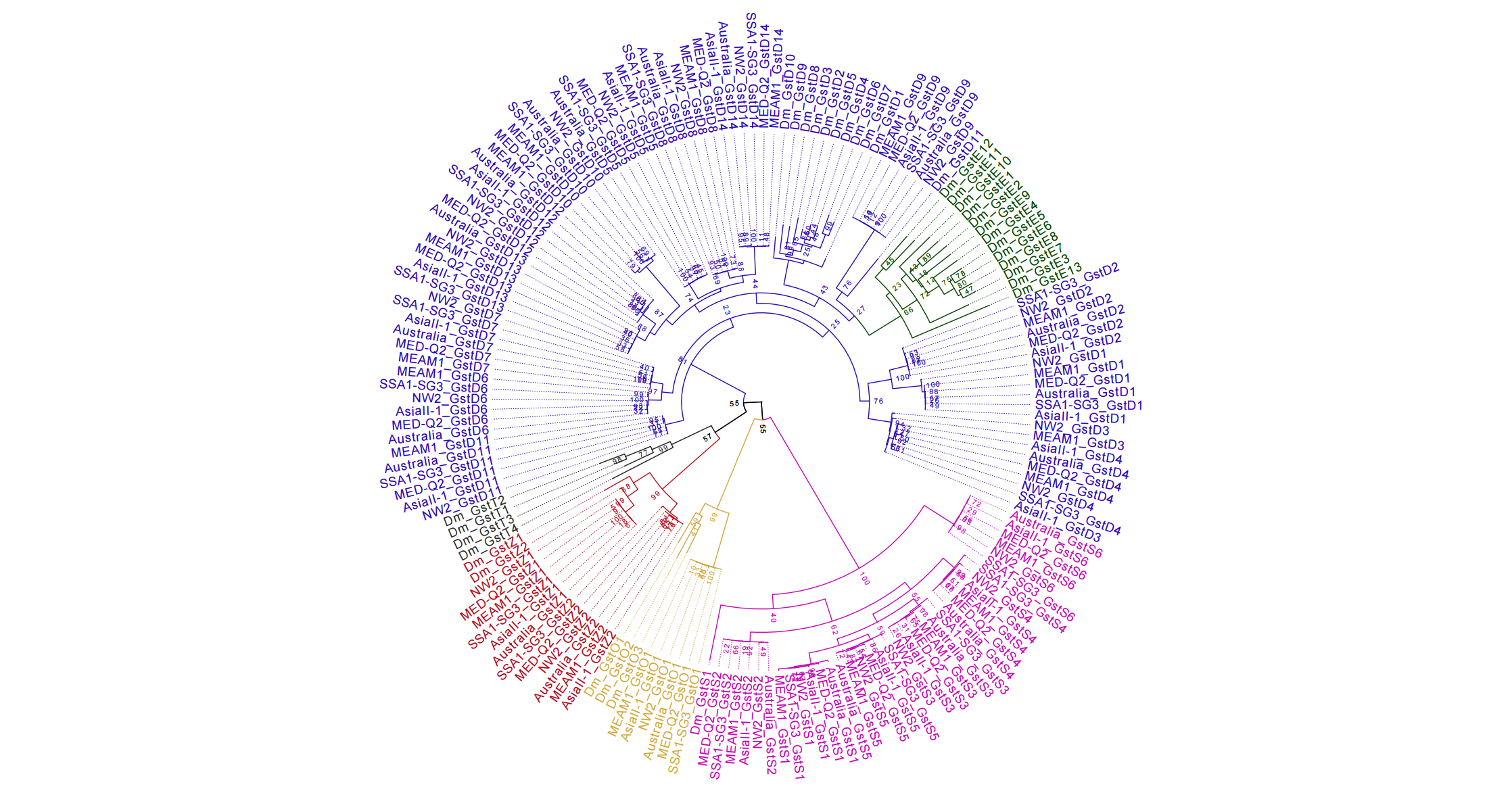

Supplement: evaa002_Supplementary_Data [file evaa002_supplementary_data.zip › Supplementary Figure S2 with legend.docx]
