# Supplementary material for: Molecular Evolution of the Glutathione S-Transferase Family in the Bemisia tabaci Species Complex
Source: Genome Biol Evol. 2020 Jan 23;12(2):3857–72. doi: 10.1093/gbe/evaa002 (PMC7058157; doi:10.1093/gbe/evaa002)
Supplement: evaa002_Supplementary_Data [file evaa002_supplementary_data.zip › Supplementary tables S1_S4 revised version.docx]

**Supplemental Table S1 - Primers for PCR amplification of the 25 GST genes in *B. tabaci* and their melting temperature.**

| **Tm (C^O^)** | **Forward primer** | **Reverse primer** | **species** | **Gene** |
| --- | --- | --- | --- | --- |
| 55 | CGAAGCTATAAATCGTAATGG | GTAATGCTAAAACTGCAATCA | All | GstS3 |
| 50 | CCAGATTATTCAGATTCAAGAGGATG | ATCGCCAAACCTGAGGTCAC | All | GstD9 |
| 56 | CGTTTGGCGAGTTGTAATTTGCGG | CTTATTTGAGAATATTGATTCTGTTACGAG | MEAM1  ASIA II-1  NW2 | GstD3 |
| 50 | CGTTTGGCGAGTTGTAATTTGCGG | CTTATTTGAGAATATTGATTCTGTTACGAG | All | GstD4 |
| 65 | CAGTTTTTACAGTGACGCAGCATG | CTGCAGTAAATGGCAAACATCGTTTA | All | GstS2 |
| 65 | GGATATCAACTTCTCTCAGCATG | GATGCTTGATCACCTGAATATTA | All | GstZ1 |
| 66 | GCAGCGTTGCTTCCCGTGC | GATGTTACAGCGGCGTGTCTCTTTA | All | GstD1 |
| 57 | GTTTACATTAAGGAAACATGACGAT | TTATTGCAATTTCCGAACTGC | SSA1-SG3 | GstD5 |
| 57 | CGTCAATCGGTTGACAGTGAACG | TTATGCTTATGTAGTGAGTTATTG | Rest |  |
| 60 | AGATGCTGTCTCTCACTAAGC | TCTCTTCTTCTTCAAGTTCTTCATTTTTATCA | ASIA II-1 | GstD2 |
| 60 | GGCCGATTCAACGTTTCGTTCC | CTCTTTCTTCAAGTCCTTCATTTTTATCA | Rest |  |
| 64 | CCATGGCTCCTCCAAAACTAACG | TGCCGAACCTACAATCACCAATC | All | GstS1 |
| 57 | GGTGCTGAGATCGGGACAGG | GTGCTAAAACTTAACAAATGTCAAC |  | GstS4 |
| 58 | CACGATGGCTCCTCCCAAG | TAATTTGATCACCAGTCGGTTACC | SSA1-SG3 | GstS5 |
| 60 | ATTGCAGTGAAAAATCACGATGGC | CATGTATTATTCAAATAATTCGATCACC | Rest |  |
| 60 | TGTTCCCTGGGGATTTTACTATG | GACGTAAGCGGCAACAATCTA | Australia | GstD13 |
| 57 | GTTCCCTGGAGATTTCACTATG | ACGCAAGTGGCATCAATCTA | rest |  |
| 55 | TTCAGAGCATCACAATGCCG | TTATTCATAGTCTTTATTTTGAGATGATT | All | GstD14 |
| 52 | CCAAAAAGCGCACACGATG | AACAATGCCTGTTGCTTTTA | All | GstD7 |
| 57 | ATGTCCGGGAAAGAGGYGGC | TATTTAGCATCTTTGACAGCGTCAGG | All | GstZ2 |
| 65 | GTGACTCGGATGATGCCATT | ACCGACTAAGCATTCTTCCG | SSA | GstD12 |
| 60 | CGTAAGTCAGGATTGGTGCTGTC | GAGGACACGCGAAGTATCCGACTA | Rest |  |
| 60 | CTGCGATAGTTCGTTACACCTACATG | AGCTGGAAACTTCTCTCAACTCGG | All | GstD11 |
| 65 | GAAACTTCGTCAGCCCACAATG | CTTTTGAACCTTTTGAAGCTTGATTA | All | GstO1 |
| 63 | GATCCCATCCGGACCGCGG | AGATTCGAAATGATATGAGACTATGACTCTCA | MEAM1 NW2 MED-Q1 Australia | GstD10 |
| 56 | ATGACCATCGACTTGTATCAT | TCATTTTGACTTTAAATCTTCCCA | All | GstD6 |
| 55 | GTTGTGAAAGATCGGAAATATG | TGTTAAGTCATTGAGCCGTCTA | All | GstMicrosomal1 |
| 60 | CCAGTGCATTCACCTACCGC | GGTGGGAACTTAAGAGAATATCTTA | All | GstD8 |
| 64 | CTAATTGTAATATTCCGAGGCTGGTCTC | AGCAGATGGCACCCTCCTAC | SSA | GstS6 |
| 65 | GCGTTTCCGTAGAGGATCCACC | GACCATTATCTTCGTGAGCAAGCAG | Rest |  |
| 64 | CCCTAAAGTTAGAGGATTTGTAACTCGAG | TGAGAAAATGAGTTCGATAATGGGAAGG | All | GstMicrosomal2 |

**Supplemental Table S2 – Quality scores of the structural models.** The scores were obtained from the I-TASSER output. The TM and RMSD scores were computed by comparisons to the model used by the server.

| **Protein** | **Species** | **Presented in: figure, color, (residues)** | **C-score** | **TM-score** | **RMSD** |
| --- | --- | --- | --- | --- | --- |
| **GstD14** | MEAM1 | Fig5, grey (27, 183)  Fig6, grey (111) | 1.29 | 0.89±0.07 | 3±2.1 |
| **GstD14** | Australia | Fig5, off-white (27, 183)  Fig6, off-white (111) | 1.32 | 0.90±0.06 | 2.9±2.1 |
| **GstZ2** | SSA1-SG3 | Fig5, off-white (134)  Fig6, off-white (117 ,118, 134) | 1.16 | 0.87±0.07 | 3.2±2.3 |
| **GstZ2** | MED-Q1 | Fig5, grey (134)  Fig6, grey (117 ,118, 134) | 1.04 | 0.86±0.07 | 3.4±2.4 |
| **GstD6** | MEAM1 | Fig5, off-white (126) | 1.25 | 0.89±0.07 | 3.0±2.2 |
| **GstD6** | MED-Q1 | Fig5, grey (126) | 1.27 | 0.89±0.07 | 3.0±2.1 |
| **GstD5** | NW2 | Fig5, grey (28) | 1.27 | 0.89±0.07 | 3.0±2.2 |
| **GstD5** | MEAM1 | Fig5, off-white (28) | 1.13 | 0.87±0.07 | 3.3±2.3 |
| **GstD10** | MED-Q1 | Fig5, off-white (1, 28, 128, 171, 185)  Fig6, grey (90) | 0.65 | 0.80±0.09 | 4.2±2.8 |
| **GstD10** | SSA1-SG3 | Fig5, grey (1, 28, 128, 171, 185)  Fig6, grey (104, 105) | 1.24 | 0.88±0.07 | 3.0±2.2 |
| **GstD10** | NW | Fig6, off-white (90, 104, 105) | 0.74 | 0.81±0.09 | 4.0±2.7 |
| **GstD12** | MEAM1 | Fig5, off-white (27, 47)  Fig6, off-white (111, 115, 164) | 1.27 | 0.89±0.07 | 3.0±2.2 |
| **GstD12** | SSA1-SG3 | Fig5, grey (27, 47)  Fig6, grey (111, 115, 164) | 1.02 | 0.85±0.08 | 3.5±2.4 |
| **GstD7** | MEAM1 | Fig6, grey (200, 202, 204, 205) | 1.30 | 0.89±0.07 | 2.9±2.1 |
| **GstD7** | SSA1-SG3 | Fig6, off-white (200, 204, 205) | 1.32 | 0.90±0.06 | 2.9±2.1 |
| **GstD7** | NW2 | Fig6, off-white (202) | 1.33 | 0.90±0.06 | 2.9±2.1 |
| **GstS4** | NW2 | Fig6, off-white (107, 163) | 1.41 | 0.91±0.06 | 2.6±1.9 |
| **GstS4** | SSA1-SG3 | Fig6, grey (107, 163) | 1.41 | 0.91±0.06 | 2.6±1.9 |
| **GstD8** | MEAM1 | Fig6, grey (108) | 1.26 | 0.89±0.07 | 3.0±2.2 |
| **GstD8** | SSA1-SG3 | Fig6, off-white (108) | 1.28 | 0.89±0.07 | 3.0±2.1 |
| **GstD4** | Asia II-1 | Fig6, grey (109) | 0.93 | 0.84±0.08 | 3.7±2.6 |
| **GstD4** | SSA1-SG3 | Fig6, pink (109) | 0.97 | 0.85±0.08 | 3.7±2.5 |
| **GstD4** | MED-Q1 | Fig6, off-white (109) | 1.06 | 0.86±0.07 | 3.5±2.4 |
| **GstD3** | MEAM1 | Fig6, off-white (112, 116) | 0.88 | 0.83±0.08 | 3.8±2.6 |
| **GstD3** | Asia II-1 | Fig6, grey (112, 116) | 0.76 | 0.82±0.09 | 4.1±2.7 |

**Supplemental Table S3 – Significance values of positively selected residues in different tests.** Significant test results are displayed in bold. The output of CODEML does not provide BEB probability for residues with no probability, therefore no values are presented for those residues. In black – results for protein-based phylogenies, in red - results for nucleotide-based phylogenies *(see section positive selection tests, Material and Methods*). In all outlined residues, at least one positive selection test passed the significance cut-off using both the protein- and nucleotide-based phylogenies.

| **Residue** | **MEME**  **P - value** | **BUSTED**  **Likelihoods of:**  **Unconstrained**  **Constrained** | **CODEML***  **Foreground branch for M2A**  **Models likelihood**  **BEB probability (model)** | **FEL**  **P - value** | **REL**  **Bayes Factor**  **{dN>dS}** |
| --- | --- | --- | --- | --- | --- |
| **GstMicrosomal1 132** | **0.02**  **0.02** | -16.7  -16.44  - | No probability  No probability | **0.033**  0.28 | 1  1 |
| **GstZ2 134** | **0.043**  **0.04** | **-19.12**  **-23.35**  **-20.98**  **-23.24** | No probability  No probability | 0.147  0.113 | 1  1 |
| **GstD5 28** | **0.023**  **0.02** | -13.78  -13.8  -13.77  -14.01 | NW2  M2A_0_ -1725.20, M2A_A_ -1724.57  M7 -1793.83, M8 -1793.63  **0.99 (M2A_0_)**  M2A_0_ -1725.20, M2A_A_ -1724.58  M7 -1726.2, M8 -1726.2  **0.99 (M2A_0_)** | 0.34  0.066 | 1  1 |
| **GstD10 1** | 0.062  0.06 | **-23.17**  **-28.01**  **-21.4**  **-23.89** | SSA1-SG3  **M2A_0_ -2153.59, M2A_A_ -2148.89**  **M7 -2154, M8 -2145.4**  **0.98 (M8)**  SSA1-SG3  **M2A_0_ -2153.89, M2A_A_ -2149.16**  **M7 -2154.23, M8 -2145.51**  **0.98 (M8)** | 0.075  0.094 | **113.5**  19.388 |
| **GstD10 28** | 0.14  0.67 | **-19.01**  **-21.39**  -21.95  -23.41 | SSA1-SG3  **M2A_0_ -2153.59, M2A_A_ -2148.89**  **M7 -2154, M8 -2145.4**  **0.965 (M8)**  SSA1-SG3  **M2A_0_ -2153.89, M2A_A_ -2149.16**  **M7 -2154.23, M8 -2145.51**  **0.965 (M8)** | 0.89  0.93 | 11.45  33.19 |
| **GstD10 113** | **0.018**  **0.02** | **-24.69**  **-29.16**  **-25.31**  **-28.25** | SSA1-SG3  **M2A_0_ -2153.59, M2A_A_ -2148.89**  **M7 -2154, M8 -2145.4**  **0.99 (M8)**  SSA1-SG3  **M2A_0_ -2153.89, M2A_A_ -2149.16**  **M7 -2154.23, M8 -2145.51**  **0.991 (M8)** | **0.021**  **0.38** | **92.44**  **119.22** |
| **GstD10 171** | 0.63  0.28 | **-29.42**  **-32.26**  -29.78  -31.59 | SSA1-SG3  **M2A_0_ -2153.59, M2A_A_ -2148.89**  **M7 -2154, M8 -2145.4**  **0.98 (M8)**  SSA1-SG3  **M2A_0_ -2153.89, M2A_A_ -2149.16**  **M7 -2154.23, M8 -2145.51**  **0.986 (M8)** | 0.5  0.875 | **144.77**  **117.52** |
| **GstD10 185** | 0.069  0.11 | **-22.57**  **-26.36**  **-22.53**  **-25** | SSA1-SG3  **M2A_0_ -2153.59, M2A_A_ -2148.89**  **M7 -2154, M8 -2145.4**  **0.97 (M8)**  SSA1-SG3  **M2A_0_ -2153.89, M2A_A_ -2149.16**  **M7 -2154.23, M8 -2145.51**  **0.966 (M8)** | **0.049**  0.259 | **127.78**  **56.51** |
| **GstD12 27** | **0.018**  **0.02** | **-15.42**  **-18.9**  **-15.44**  **-18.9** | NW2, ASIA II-1, SSA1-SG3, Australia  **M2A_0_ -2175.87, M2A_A_ -2170**  M7 -2170.58, M8 -2170.58  **0.996 (M2A_0_)**  NW2, ASIA II-1, SSA1-SG3, Australia  **M2A_0_ -2175.87, M2A_A_ -2170.62**  M7 -2170.58, M8 -2170.58  **0.996 (M2)** | 0.73  - | 1  1 |
| **GstD12 47** | **0.0015**  **0.01** | **-16.54**  **-27.96**  **-16.6**  **-27.18** | No probability  NW2, ASIA II-1, SSA1-SG3, Australia  **M2A_0_ -2175.87, M2A_A_ -2170.62**  M7 -2170.58, M8 -2170.58  **0.996 (M2A_0_)** | 0.18  0.37 | 1  1 |
| **GstD14 27** | **0.012**  **0.04** | **-19.8335**  **-32.266**  **-19.73**  **-32.27** | No probability  No probability | 0.38  0.181 | 0.77  0.79 |
| **GstD14 183** | **0.039**  0.67 | **-15.7234**  **-20.4021**  **-15.63**  **-20.49** | No probability  MED-Q2, MEAM1, SSA1-SG3  **M2A_0_ -1725.99, M2A_A_ -1719.69**  M7 -1723.99, M8 -1722.78  0.828 (M2A_0_) | 0.082  0.127 | 0.000331  0.000354 |

| **Gene** | **Iss.c** | **Iss** | **Iss 95% lower limit** | **Iss 95% upper limit** |
| --- | --- | --- | --- | --- |
| **GstD5** | 0.7653 | 0.1512 | 0.1259 | 0.1765 |
| **GstD14** | 0.7658 | 0.1692 | 0.1394 | 0.1991 |
| **GstD12** | 0.7653 | 0.2439 | 0.2092 | 0.2787 |
| **GstZ2** | 0.7644 | 0.1728 | 0.1460 | 0.1996 |
| **GstMicrosomal1** | 0.7489 | 0.1223 | 0.0943 | 0.1503 |
| **GstD3** | 0.8056 | 0.2959 | 0.2630 | 0.3287 |
| **GstD4** | 0.7662 | 0.2220 | 0.1922 | 0.2519 |
| **GstS3** | 0.7619 | 0.1737 | 0.1447 | 0.2027 |
| **GstD9** | 0.7666 | 0.2490 | 0.2086 | 0.2893 |
| **GstS2** | 0.7817 | 0.1762 | 0.1472 | 0.2051 |
| **GstZ1** | 0.7738 | 0.0898 | 0.0687 | 0.1109 |
| **GstD1** | 0.7799 | 0.2453 | 0.2143 | 0.2763 |
| **GstD2** | 0.7736 | 0.2157 | 0.1887 | 0.2427 |
| **GstS1** | 0.7619 | 0.1765 | 0.1487 | 0.2043 |
| **GstS4** | 0.7617 | 0.1738 | 0.1452 | 0.2023 |
| **GstS5** | 0.7709 | 0.1934 | 0.1635 | 0.2234 |
| **GstD13** | 0.7658 | 0.2304 | 0.1982 | 0.2625 |
| **GstD14** | 0.7642 | 0.1540 | 0.1283 | 0.1796 |
| **GstD7** | 0.7644 | 0.1739 | 0.1483 | 0.1995 |
| **GstD11** | 0.7828 | 0.2460 | 0.2151 | 0.2768 |
| GstO1 | 0.7694 | 0.1016 | 0.0811 | 0.1220 |
| **GstD10** | 0.7823 | 0.2679 | 0.2328 | 0.3031 |
| **GstD6** | 0.7732 | 0.2109 | 0.1816 | 0.2401 |
| **GstD8** | 0.7738 | 0.1405 | 0.1152 | 0.1659 |
| **GstS6** | 0.7614 | 0.1769 | 0.1511 | 0.2027 |
| **GstMicrosomal2** | 0.7495 | 0.1238 | 0.0946 | 0.1531 |

**Supplemental Table S4 – Measurements of substitution saturations.**
